# The Silent Spill: A Case Report on Navigating the Challenges of a Chyle Leak

**DOI:** 10.7759/cureus.62072

**Published:** 2024-06-10

**Authors:** Prachi Surolia, Rajanikanth Kambala, Nitin Bhola, Anchal Agarwal

**Affiliations:** 1 Oral and Maxillofacial Surgery, Sharad Pawar Dental College and Hospital, Datta Meghe Institute of Higher Education and Research, Wardha, IND

**Keywords:** intraoperative assessment, postoperative management, neck dissection, carcinoma, chyle leak

## Abstract

A chyle leak occurs due to a discontinuity in the thoracic duct. It is a very rare condition that occurs as a result of injuries or surgical procedures. Chyle is rich in antibodies. Its functions are to maintain the equilibrium of the human fluid system, draw in fatty acids, and maintain the natural immunity of humans. It is identified by the increased quantity of drains, which show a milky white color and clinically palpable supraclavicular collection. It is a condition that has to be managed as soon as possible as it leads to serious nutritional debridement, electrolyte imbalance, and complications such as chylothorax and chylomediastinum. It is managed by various surgical and conservative approaches, such as ligating the thoracic duct, using sclerosing agents, giving total parenteral nutrition, and restricting physical activities, as discussed in this article.

## Introduction

The word chyle originated from the Greek word “Chylos,” a synonym for juice. It occurs when there is a break in the continuity of the thoracic duct and the milky, lipid-rich chyle leaks to the nearby tissues. By the end of the sixth week of intrauterine life, stem cells begin to differentiate into lymphatic channels. A plexus is formed when several lymphatic clefts ultimately unite to form one. The right lower half and the entire left half of the body below the collarbone have a single duct that drains the lymphatic fluid as a result of the selective atrophy of these plexuses [1]. Chylomicrons, a mixture of phospholipids, cholesterol esters, and long-chain triglycerides, comprise chyle. There are a lot of lymphocytes in it, mostly T lymphocytes, which range in number from 400 to 6800 cells.

Plasma and chyle have comparable electrolyte concentrations and have abundant immunoglobulins along with fat-soluble vitamins. Its incidence ranges from 1% to 5% in neck dissection [2]. More frequently, this happens when the left side is dissected [2-4]. Chyle leak causes wound dehiscence, a protracted healing period for wounds, systemic metabolic imbalance, extended hospital stays, and a delay in starting adjuvant therapy. Early, proper diagnosis and therapy are essential for a successful surgical outcome. Although chyle leaks are uncommon, they can have serious consequences, and they can be managed conservatively or by surgical modalities.

## Case presentation

**Figure 1:**
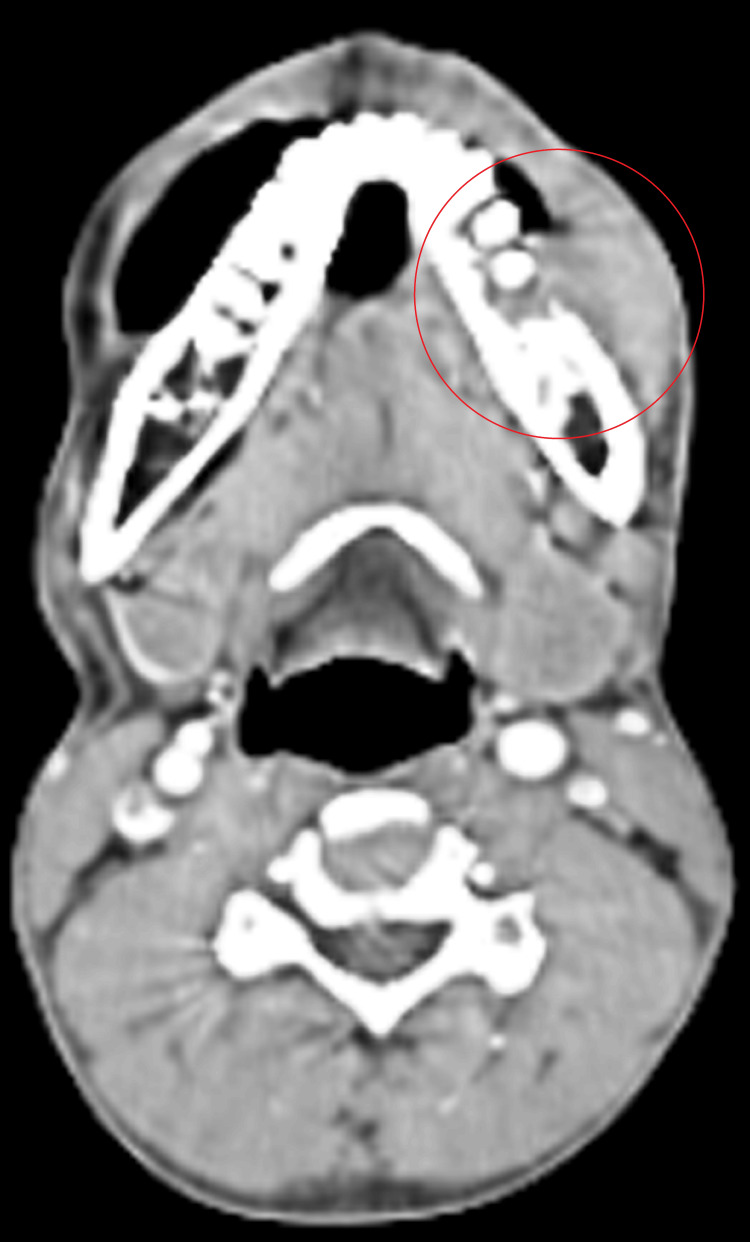
Computed tomography revealing a lesion in the left lower gingiva buccal sulcus.

A 34-year-old male reported to our cancer center and gave a history of a non-healing ulcer over the lower left back region of his jaw for 9-10 months. Upon local examination, a non-healing ulcer of 3 x 4 cm was observed, extending from the first to the third mandibular molars, along with the loss of alveolar bone on the left side. A neck examination revealed palpable bilateral submandibular and submental lymph nodes. The histopathological diagnosis was a moderately differentiated squamous cell carcinoma of the lower left gingivobuccal sulcus. The computed tomography (CT) report also revealed submental, bilateral submandibular, and bilateral jugulodigastric lymph nodes. The CT scan also displayed a heterogeneously enhancing irregular soft tissue density lesion in the lower left gingivobuccal sulcus, anteriorly up to an angle of the mouth and the left parasymphyseal region. It also extends posteriorly to the ramus of the mandible on the left side, reaching up to the skin (Figure 1).

After the discussion with our surgical oncology team, the surgical plan involved a composite resection of the lesion (segmental mandibulectomy from the canine tooth of the right side to the subcondylar region on the left side), bilateral modified radical neck dissection type III, and a reconstruction. A tracheostomy was also performed.

**Figure 2:**
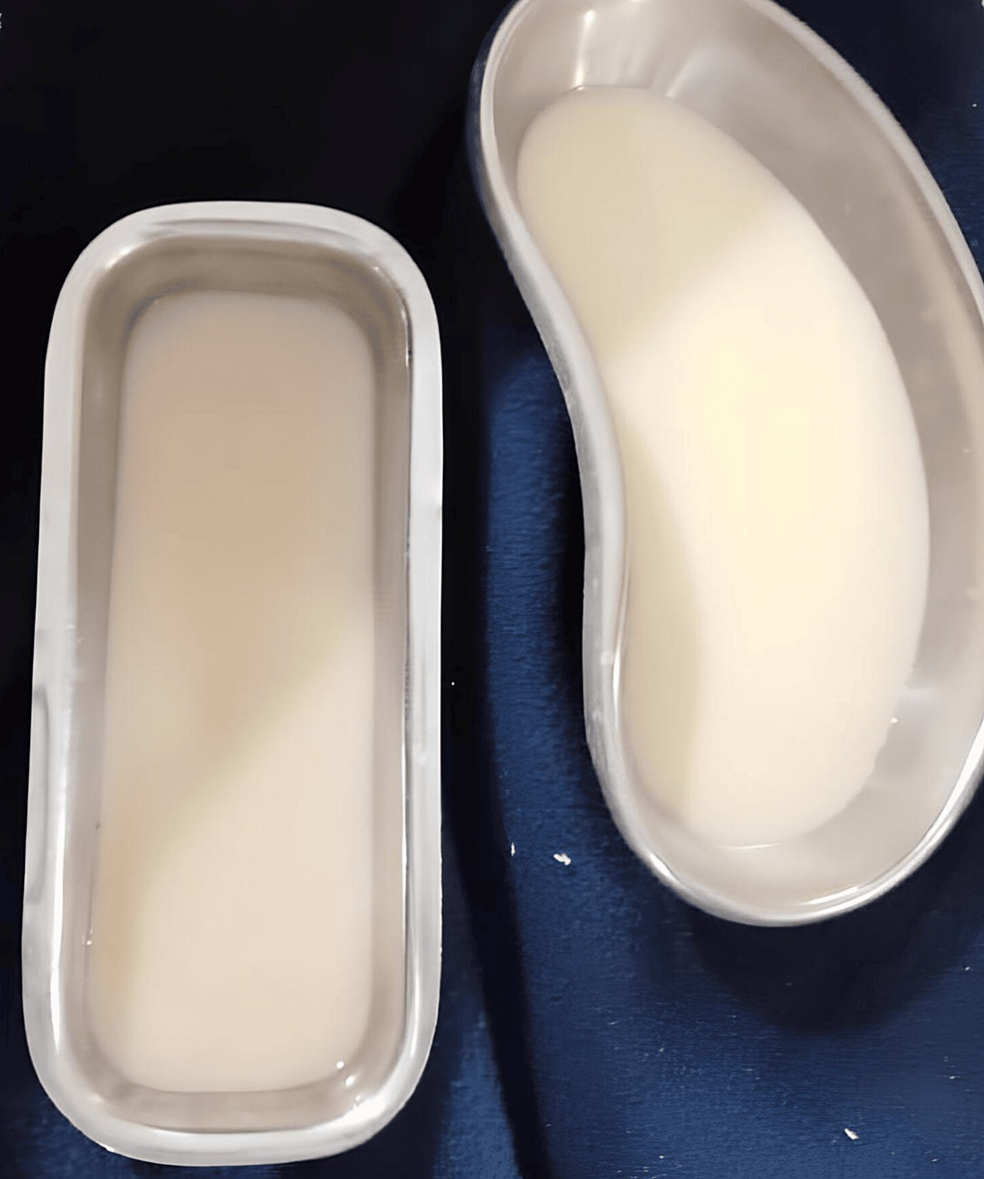
Milky chyle fluid collected from the neck drain.

Intraoperatively, a left-sided chyle leak was observed following lower internal jugular chain (deep cervical) node dissection. This was checked by Trendelenburg position and Valsalva maneuver, following which clear shiny fluid was seen. After diagnosis, repair of the duct was performed intraoperatively with loupes magnification. The source of the chyle leak was identified and was secured with a permanent suture (5/0 Prolene). The Valsalva maneuver was again performed to cross-check, and no shiny fluid was present. The patient experienced a smooth recovery during his first day after surgery in the intensive care unit (ICU), with a total of 150 mL of fluid drained from both neck drains. Feeding was started through a nasogastric tube. By the second day after surgery, 170 mL of serosanguinous drains were present. By the third day, the patient remained in satisfactory health, but there was a buildup of 250 mL of milky fluid in the left neck drain (Figure 2).

A sample of this fluid was forwarded for biochemical analysis to verify the presence of triglycerides. A diagnosis of chyle was made as triglyceride levels were 220 mg/dL (which is more than the normal levels of 100 mg/dL). On postoperative day four, he drained 400 mL of fluid. After arriving at the diagnosis, enteral feeding was stopped. To avoid increasing intrathoracic pressure, the patient was kept immobilized, and neck drains were kept passive. The administration of octreotide, a somatostatin analog, started with a subcutaneous injection of 100 µg every eight hours; it facilitated the activation of somatostatin receptors and modified the splanchnic arteriolar resistance and blood flow in the gastrointestinal system, resulting in a decrease in chyle flow. Additionally, to prevent any increase in chyle leak, a pressure dressing was given on level IV nodes. Dietary modification, such as a low-fat diet and a calorie requirement of 2000 kcal/day, was advised.

On day five, the patient's left neck drain was 160 mL, and the right neck drain was 130 mL. On day six, the right neck drain was nil, and the left neck drain was 90 mL. On days seven and eight, 30 mL and 10 mL of drains were collected, respectively. On day nine, there were no further occurrences of chyle leakage or neck collections. Throughout the entire course of events, vital signs, fluid balance, electrolytes, and liver function tests were closely monitored. The patient continued to recover without complications and was discharged in good health.

## Discussion

Anatomy

**Figure 3:**
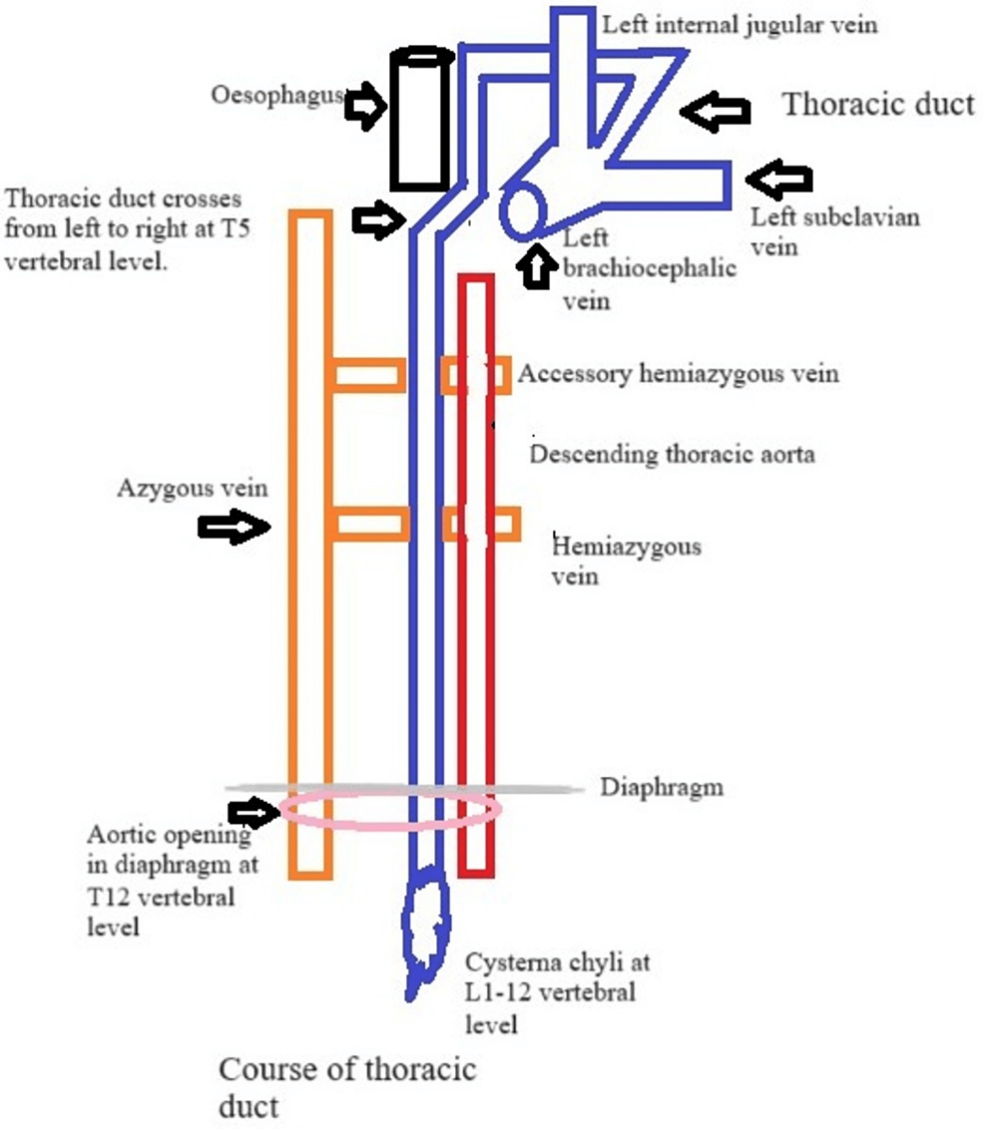
Course of the thoracic duct.

The lymphatic system comprises lymphatic capillaries, veins, tonsils, spleen, thymus, nodes, ducts, and other bodily structures running alongside blood vessels to circulate lymph throughout the body. The thoracic duct is the largest duct of the lymphatic system that serves as the primary route for draining lymph from various regions, including the lower limbs, kidney, urinary bladder, wall of the abdomen, organs of the abdomen, the breast, the left side of the head and neck, the left lung, the left forearm, the left wall of the thorax, and the left part of the chest. It starts at the top of the cisterna chyli and terminates at the angle of the vein on the left side (Figure 3).

Its trajectory consists of the abdomen part, thoracic part, and cervical segments, with the abdominal part situated in the posterior to the peritoneum space and the posterior mediastinum containing the thoracic part. As it progresses, the thoracic duct curves to the left, passing beside the left laryngeal nerve near the esophagus, crossing the arch of the aorta, moving through the superior thoracic aperture, climbing behind the left subclavian vein and arriving at the lower neck [5]. At the C7 level in the neck, the thoracic duct turns outward and forward, forming an arc within the triangle formed by the vertebrae artery. This particular triangle of the neck is defined by the subclavian artery, anterior longus colli muscle, and scalene muscle. Within this region, the duct transits from medially to laterally, passing behind the left internal jugular vein, left vagus nerve and vein, and left common carotid artery before ultimately entering the venous angle [5,6]. The lymphatic duct of the right side is smaller and transports lymph exclusively from the right half of the mediastinum, right side of the chest, right arm, right breast, right lung, right head, and neck and empties into the right venous angle [5].

Physiology

The lymphatic system regulates the equilibrium of fluid composition, the drawing of fatty acids in the body, and immunity. The large quantity of proteins and interstitial fluid from the tissues that are not able to be transported back via the blood vessels are carried by the lymph system. Lymphatic circulation is improved through pumping, which can occur due to external or neighboring tissue motion or internal contractions of specific muscles within lymphatic vessel linings [7].

The lymphatic system plays a crucial role in regulating and supporting intestinal functions. It aids in lipid transportation, immune defense against infections, and the elimination of excess fluids. Within the small intestine villi, there are lymph capillaries called lacteals that are responsible for absorbing fats and fat-soluble vitamins. These lacteals produce a lipid-rich fluid known as chyle, which appears milky white and contains chylomicrons, which are specialized lipoproteins that facilitate fat transport in a watery environment. The chyle from the intestines is directed specifically to the cisterna chyli. This chyle comprises a fluid resembling blood plasma, proteins (2%-4.5%), large amounts of chylomicrons, glucose, trace elements, fat-soluble vitamins, white blood cells, and substantial quantities of chylomicrons [8]. Its electrolyte makeup closely resembles that of plasma. The primary lipid constituent is triglycerides. The rate of chyle flow varies based on food, peristalsis, digestive activity, physical activity, respiratory actions, coughing, changes in intrathoracic and intra-abdominal pressures, and respiratory actions [2,9].

Pathophysiology

Disruption of the lymphatic system causes a buildup of proteins in the spaces between cells, leading to edema. When there is a leak of chyle and the loss of its components, it can result in severe malnutrition, imbalances in electrolytes and fluids, and other systemic complications that can be life-threatening. This loss of fat, electrolyte, and protein causes primary hypoalbuminemia, low sodium levels, low potassium levels, and low calcium levels. Hypoalbuminemia can worsen primary hypovolemia caused by loss of fluids and change in fluid distribution within the body. Moreover, a chyle leak can result in the depletion of white blood cells, which leads to a weakened immune system, poor wound healing, wound infections, sepsis, and fistula.

Chyle fistulas, which occupy space, exert pressure on adjacent structures. This increase in pressure due to accumulation leads to inadequate blood supply to the tissue flap, encouraging tissue necrosis in the flap [10]. The outcomes can vary from minor to severe conditions, such as chylothorax or chylomediastinum, which can be life-threatening [11]. These factors can result in prolonged hospitalization, increased medical expenses, and a less positive surgical result. In severe instances, it can even be fatal [12]. Chyle leak difficulty arises in 40% of patients, primarily causing imbalances in electrolytes and proteins, particularly hyponatremia and hypoalbuminemia [13].

Intraoperative evaluation

During surgery, if a leak is detected, immediate action must be taken to halt it. There should be a special focus on meticulously examining the venous angle region, especially following head and neck procedures that involve dissection of level lower jugular (deep cervical) nodes. If a lymph is seen during surgery, it is important to identify its origin. Utilizing surgical loupes or an operative microscope can aid in visualizing the lymph, as identifying it can otherwise be challenging due to the variable course and collapsibility of the thoracic duct. Applying the Trendelenburg position to the patient and elevating intrathoracic pressure can assist in pinpointing the leak's source [10].

Postoperative evaluation

After surgery, a chyle leak is suspected if there is a significant buildup of milky white collection in the drains beyond what is expected or if an unanticipated surge in drains following feeding is present. Additionally, signs such as lymphedema or erythema in the neck area may be observed, and there may be a palpable collection of fluid in the supraclavicular region [14]. Usually, these signs are adequate for making a diagnosis. Nonetheless, a biochemical laboratory investigation may be helpful for arriving at a diagnosis if there is any doubt. A triglyceride crossing >100 mg/dL or a triglyceride level higher in the drain than in the serum can confirm the presence of chyle leakage [15].

Chylomicrons seen in the neck drain and lipid analysis may signify a chyle leak, but they could also be the result of tissue of the adipose layer breaking down in the incision. Triglyceride ranges are more specific and indicative in confirming a chyle leak than cholesterol levels, which might also be a cause for concern [16]. The portable SD Lipido Care test equipment (SD Biosensor, Suwon, South Korea) is useful for quickly and precisely accessing chyle leakage, according to the research conducted by Lee et al. [12]. In only three minutes, this portable device can measure a variety of lipid profiles, including total cholesterol, triglycerides, high-density lipoprotein, and low-density lipoprotein. This is accomplished by using a tiny pipette to introduce aspirated fluid from neck drainage into the equipment [12].

Management

There are multiple surgical and conservative approaches suggested for halting chyle leaks [2,3,6,17]. Nearly all authors advocate for a conservative approach as the initial treatment option, irrespective of the volume of the leak. Switching to a low-fat diet that is high in medium-chain triglycerides is one measure to address this issue [18,19]. Medium-chain triglycerides are largely water-soluble and are absorbed directly into the bloodstream through the liver, bypassing the gastrointestinal lymphatic system. Another option is total parenteral nutrition, which also circumvents the gastrointestinal lymph and delivers all essential nutrients directly [20,21]. Various dietary adjustments can resolve over 70% of chyle leaks within a two-month period, and there is no significant statistical variance in their success rates [20].

Additional conservative approaches involve the use of octreotide, a prolonged-action derivative of the pancreatic lipase inhibitor orlistat/tetrahydro lipstatin and/or the hormone somatostatin. Somatostatin works by reducing the generation of lymph through the inhibition of pancreatic and gastrointestinal enzyme production [22]. Additionally, it diminishes lymph generation and slows its movement by causing the smooth muscles of the lymphatic and digestive systems to contract. Somatostatin has a brief half-life and necessitates ongoing vein delivery. Administering the long-lasting analog octreotide (100 µg/day for two to three days subcutaneously) can halt low- and medium-volume chyle leaks within five to seven days [23,24]. It is recommended that this treatment be continued for one to two days after the chyle leak has been resolved.

Few researchers report positive outcomes even in cases of increased volume chyle leaks with extended treatment lasting 30 days [2], and some recommend it as a first-line treatment [22]. Octreotide warrants cautious use, particularly in individuals with prior cardiovascular and hepatic conditions. Commonly reported mild side effects include nausea and diarrhea, while potential severe complications may consist of cholecystitis and hypoglycemia leading to gastrointestinal bleeding. On the other hand, orlistat/tetrahydrolipstatin works by deactivating pancreatic enzymes, preventing the reabsorption of cells of fat in the gastrointestinal system and inhibiting their ingress into the intestinal circulation [25].

Another conservative approach involves reducing physical exertion to alleviate pressure on the lymph [26]. Managing coughs with antitussive medications and regulating bowel movements can prevent elevated pressure in the chest and abdomen. However, using extracorporeal compression on the neck to address lymphatic leakage, combined with removing the negative drainage system, is still a clinically uncertain approach [6].

Surgical Approach

The leakage of chyle can be detected either during the first surgery or as a complication afterward. The most effective method to halt chyle leakage is identifying and tying off the open duct during the surgery itself. However, there is no unanimous agreement on when to perform a revision surgery based on the amount of chyle lost or the timing. Some experts suggest considering revision surgery if chyle output exceeds 500 mL/day for four consecutive days, whereas others propose waiting for up to 30 days even with chyle output exceeding 1000 mL/day [2,3,6,17]. When conservative treatments are expected to be ineffective or take too much time to resolve the chyle leakage, early surgical intervention is advised due to the substantial impact of chyle leaks on nutrition and immunity [20].

For ligating the thoracic duct, it is recommended to use absorbable polyfilament sutures or non-absorbable monofilament sutures [2,3,17]. Following the ligation of the thoracic duct, the ligation should be assessed by increasing either intrathoracic or intraabdominal pressure [2,3,11]. Adding an extra muscle layer over the repaired lesion can be beneficial, with the sternocleidomastoid muscle flap being the most frequently utilized method. Local sclerosing agents such as tetracycline can also halt chyle leakage by inducing a supplementary local inflammatory reaction [27-29]. The use of topicals or delivery through a drainage system could be a potential method of administration [30,31]. Neurotoxicity is a potential complication for nearby nerves, such as the phrenic and brachial plexus. If this treatment method does not effectively halt chyle leakage, partial sclerosis could complicate subsequent surgical exploration [32]. Positive experiences after the local application of fibrin glue or polyglactin 910 Vicryl (Ethicon, Raritan, New Jersey) nets have been described in the literature [33].

If the initial neck revision surgery does not yield the desired clinical outcomes, it is reasonable to consider ligating the duct using either a transthoracic or transabdominal approach. This involves accessing the area through the right chest, either thoracoscopically or via an open thoracotomy, situated between the azygos vein and the aorta [34]. This step is highly efficient. Some experts suggest it should be the primary surgical intervention for patients experiencing leaks exceeding 1000 mL/day [8]. This procedure can worsen the patient's overall health, already compromised by extended chyle leakage. Using percutaneous thoracic duct embolization (TDE), it is feasible to check the chyle reservoir and seal it using sclerosing agents. However, it is worth noting that this procedure may occasionally necessitate multiple revisions [35]. The presence of potential anatomical variations in position and branching makes the delicate lymphatic duct prone to unintended surgical injury, leading to uncontrolled lymphatic leakage [18]. Thoracic duct injuries are commonly associated with advanced malignant metastatic diseases, particularly those affecting level IV on the left side of the neck and extending into the upper mediastinum [3]. However, concerns about chyle leakage should not restrict the appropriate oncological resection [2].

Chyle leakage typically becomes evident during the initial days after surgery, often a few hours following the patient's first meal. Its primary sign is the milky appearance of drainage fluid. On the right side of the neck, where lymph has a distinct origin and chemical makeup, an increase in the drainage of clear or blood-tinged fluid usually indicates lymphatic leakage and may appear several days after surgery. The buildup of lymph in the operated neck area manifests as a systemic metabolic imbalance and localized chemical irritation of musculoskeletal structures [6]. Loss of fluids and imbalance in electrolytes worsen the overall condition of the patient. This disruption to the patient's immune system further complicates the process of wound healing [7]. When chyle leakage reaches a volume of 1 L per 24 hours, it can become a potentially life-threatening situation. Understanding surgical anatomy is the most effective way to prevent injury to the lymphatic duct [8].

## Conclusions

Early recognition and management of this complication are of utmost importance. Intraoperatively, one should be prompt in identifying a chyle leak. If detected intraoperatively by the Valsalva maneuver, good ligation and repair should be done immediately. Postoperatively, clinicians should maintain a high suspicion index in patients with supraclavicular collection, increased drains, and surgical site flap changes. Timely implementation of early conservative and adequate measures can lead to efficient management. If required, early intervention can significantly reduce the patient's morbidity and promote good surgical site healing.
